# The Potential Therapeutic Role of Beta-Caryophyllene as a Chemosensitizer and an Inhibitor of Angiogenesis in Cancer

**DOI:** 10.3390/molecules30081751

**Published:** 2025-04-14

**Authors:** Emad A. Ahmed

**Affiliations:** Biological Sciences Department, College of Science, King Faisal University, Hofouf 31982, Alhasa, Saudi Arabia; eaahme@kfu.edu.sa

**Keywords:** Beta-Caryophyllene, chemo-sensitization, inhibitor of angiogenesis in cancer, chemoresistance, combination therapy

## Abstract

The natural, highly lipophilic bicyclic sesquiterpenes, Beta-Caryophyllene (BCP), was highlighted in several recent preclinical studies to enhance chemo-sensitization in chemo-resistant tumors and to efficiently inhibit angiogenesis and cancer cells’ ability to invade and metastasize. Previous studies have researched the reasons for the synergistic effect of Beta-Caryophyllene in combination therapy and its role as a chemosensitizer and an inhibitor of angiogenesis through investigating the involved mechanisms and signaling molecules. These include the lipophilic nature of BCP, the selective interaction of BCP with CB2, the binding affinity of BCP to the receptor binding sites at the angiogenic vascular endothelial growth factor, and the upstream effect on JAK1/STAT3 pathway and other signaling pathways. Herein, the BCP role in enhancing chemo-sensitization of chemo-resistant tumors and in inhibiting angiogenesis and cancer cells’ ability to invade and metastasize are highlighted. Beta-Caryophyllene appears to be a promising candidate in treating cancer when co-supplemented with drugs such as cisplatin, gemcitabine and sorafenib. Clinical trials are needed to validate the potential therapeutic effect of BCP as a co-supplementary drug in cancer therapy, helping to sensitize cancer response to drugs, modulating signaling pathways, and lowering the drugs’ doses besides working as anti-angiogenetic drug.

## 1. Introduction

Beta-Caryophyllene (BCP) is a highly lipophilic sesquiterpene, which can be purified from various plants like black pepper, clove, cannabis, and rosemary. It was in the mid-1990s when the antiproliferative effect of BCP has been found in human breast cancer and melanoma cells [1]. However, the anticancer characteristics of BCP and its derivative Beta-Caryophyllene oxide (BCPO) have been documented in several recent reports where BCP was found to directly inhibit cancer cells proliferation, induce cell cycle arrest, and enhance apoptosis in various types of cancers (listed in Table 1) including lung cancer [2,3,4,5] colorectal cancer [6], ovarian cancer [7], bladder cancer [8], liver cancer [9,10], pancreatic cancer [11], and breast cancer [12,13]. In the meantime, several studies suggested that BCP could be an important candidate for combined chemotherapies, enhancing the efficacy of conventional chemotherapeutic drugs, potentiating the effect of lower doses of these drugs while maintaining or improving their therapeutic impact [3,12]. Interestingly, unlike many other anticancer drugs, and probably because of its lower toxicity in normal cells, BCP seems to work as a dual modulator of oxidative stress, increasing reactive oxygen species (ROS) in cancer cells, and thus enhancing apoptosis, but reducing ROS in normal cells to protect them from damage [4,14]. However, BCP can indirectly suppress tumor invasiveness via inhibiting angiogenesis and deregulating the metastasis signaling pathways [5,6,15]. More importantly, studies, including our recent work, suggested a promising role of BCP as a chemosensitizer when co-supplemented with other drugs—it synergistically enhances the efficacy of chemotherapeutic drugs and modulates pathways involved in cancer cell survival and apoptosis [3,11,12,16,17,18]. Another important issue in BCP is its ability to bind to the body’s cannabinoid receptor 2 (CB2), where it binds selectively to the CB2 receptors and not the CB1 receptor, which makes it non-psychoactive and therapeutically appealing. The activation of the CB2 receptor by BCP can suppress pro-inflammatory cytokines production, helping to create an anti-tumor immune environment [19] and thus preventing cancer progression. Beta-Caryophyllene, a food additive approved by the Food and Drug Administration, is efficiently absorbed in the gastro-intestinal tract and can penetrate the blood–brain barrier and has a well-established safety profile, making it an attractive biomolecule for combination therapies.

## 2. Plant Species That Contain Exceptionally High Concentrations of BCP

Beta-Caryophyllene is widely found in more than 1000 types of plants, with certain plant species containing exceptionally high concentrations of BCP. Among these plants, Ylang-Ylang leaves have been found to contain the highest concentration of BCP (52%) to date [31]. This significantly exceeds the previously held assumption that black pepper is its richest source. Additionally, the leaves of the tropical tree Spondias pinnata yield 49.9% BCP [32]. The high BCP concentration in Spondias pinnata was used to study the potential for therapeutic applications, particularly in wound healing, inflammatory, and infectious conditions [33]. Another plant, Pimpinella kotschyana, a Mediterranean herb, was found to contain 49.9% BCP in its seeds [34]. This highlights the untapped potential of the herb in managing digestive disorders. Additionally, Sumac fruits contain 34.3% BCP [35], a concentration that may explain its historical use in Middle Eastern cuisine and traditional medicine, particularly for its antioxidant and anti-diabetic effects. In the meantime, black pepper, traditionally recognized for its health benefits, contains approximately 30% BCP in its fruit-derived essential oil [36], and thus, BCP is considered a key component in black pepper’s ability to enhance nutrient absorption, including compounds like curcumin. Moreover, clove buds contain 20–30% BCP in their essential oil [37], contributing to their known analgesic and antimicrobial properties. The presence of BCP in cloves further supports their role in pain management and infection prevention. Moreover, in certain cannabis strains, flowers can produce BCP concentrations of approximately 30% [38]. BCP in cannabis synergizes with cannabinoids to amplify anti-inflammatory effects, potentially enhancing the therapeutic efficacy of cannabis-based treatments. The leaves of sugar apples contain 22.9% BCP [39], supporting their traditional use in antiparasitic treatments.

## 3. Cytotoxicity of Beta-Caryophyllene in Normal and Cancer Cells

In normal, non-cancerous cells, BCP was found to exhibit selective cytotoxicity—while it is toxic to cancer cells at lower concentrations, its toxicity in normal cells occurs only at higher doses. In other words, in most cytotoxicity studies, high concentrations of BCP were required to observe a cytotoxic effects in normal cells (Table 2). For instance, BCP’s IC50 value was found to be >100 μM in skin fibroblasts [33] and peripheral blood mononuclear cells (PBMCs, [40]). However, the value was >200 μM in normal human fibroblasts [7]. Additionally, in normal human lymphocytes, BCP exhibited negligible cytotoxicity at concentrations below 100 µM, demonstrating its safety for normal tissues [41]. This observed low toxicity and minimal interference in normal cellular functions is probably due to its ability to selectively induce apoptosis and oxidative stress in cancer cells while sparing normal cells at lower concentrations. On the other hand, Beta-Caryophyllene’s I50, for many cancer cell lines typically ranges from 19 to 64 μM, depending on the context of specific experimental conditions (Table 3). For example, BCP showed significant cytotoxicity in two different human bladder cancer (BC) cell lines, T24 and 5637 cells; the effect was dependent on the concentration and exposure time, and the IC50 value was 40 µg/mL [8]. Additionally, IC50 values of BCP were found to be around 19 µM for HCT-116 (colon cancer), 20 µM for MG-63 (bone cancer), 27 µM for PANC-1 (pancreatic cancer) cells [6,42]. Similarly, the mean IC50 values of BCP administered as pure compound were 19.2 and 44.7 µg/mL in MDA-MB-468 and HepG2 cells, respectively [23]. In the meantime, BCP displayed an obvious selective effect in hepatoma cells (IC50 = 63.7 μg/mL) when compared with normal fibroblasts (IC50 = 195.0 μg/mL), in which lower cytotoxicity of BCP was observed [43]. In agreement with that, although higher doses of BCP were required to enhance cytotoxicity in cells such as ME-180 (cervical cancer) and MCF-7 (triple negative breast cancer cells), BCP doses needed to induce toxicity in normal cell lines such as 3T3-L1 and RGC-5 were at least two folds higher, relative to those for cancer cells [44]. In all, and based on the above overview of BCP’s cytotoxic effect in normal and cancer cells, BCP may selectively target cancer cells over normal ones, potentially allowing for an effective cancer treatment with reduced side effects.

## 4. Beta-Caryophyllene Enhances Oxidative Stress and Activates Cell Cycle Checkpoints to Promote Apoptosis in Cancer Cells

Cell cycle arrest induction and triggering apoptosis in cancer cell lines treated with BCP was reported to occur through upregulating reactive oxygen species (ROS), inducing mitochondrial dysfunction, activating caspases and Bax, or downregulating Bcl-2 in these cells. In the meantime, disruption of DNA replication and DNA damage response (DDR) are critical mechanisms by which certain anticancer agents, including natural compounds, like BCP, induce cell cycle arrest and promote apoptosis in cancer cells. In this regard, recently, BCP was found to delay DNA damage repair and to reduce the levels of pERK, pAKT, and NF-κB, leading to the increase of apoptosis and reduction of cancer cell survival/proliferation rate [18]. Similarly, a cell cycle analysis indicated that BCP enhanced S-phase arrest in ovarian OAW-42 cells that displayed a high rate of apoptosis mediated by cleavage of PARP and caspase-3 [7]. Additionally, in breast cancer MDA-MB-468 cells, BCP activated checkpoint that enhanced the induction of G2/M phase arrest, and thus promoted cell death [13], which agrees with what has been obtained in doxorubicin treated cholangiocarcinoma cells (CCAs) supplemented with BCP [46]. In line with that, BCP induced G1 cell cycle arrest via upregulating p21 and p27 and downregulating cyclin D1 and E, and other cyclin-dependent protein kinases in human lung cancer cells [2]. Similar effect has been found on lung adenocarcinoma, where BCP enhanced the apoptosis through blocking the G2/M and S phases [48]. In agreement with that, BCP-treated A549 cells displayed a significant increase in the caspase’s levels (3, 7 and 9), while the Bcl-2 was significantly downregulated [4]. On the other hand, BCP acts via CB2 receptor signaling to trigger apoptosis in several cancer cells, such as the aggressive tumor, glioblastomas, that is characterized by uncontrolled inflammatory response and cell proliferation. BCP’s ability to induce anti-inflammatory effect via interacting with CB2 and modulating peroxisome proliferator-activated (PPARg) receptor using two vitro models of glioblastoma was associated with a significant anti-proliferative effect via reducing the cells’ viability, blocking cell cycle, and increasing the apoptosis rate [48]. Similarly, in MM cells, BCP inhibited cell proliferation through modulating critical signaling pathways like Wnt/β-catenin, AKT and cyclin D1/CDK [49]. However, BCP inhibited non-small cell lung cancer (NSCLC) growth via dysregulating the antioxidant enzymes (SOD, CAT and GPx) and ROS, and increasing apoptotic factors (cleaved caspase-3 and Bax) in these cells [20]. In the same line, BCP suppressed the tumor incidence by downregulating inflammation and inducing caspase-3-mediated apoptosis in liver carcinoma through the mitigation of oxidative stress markers (MDA, NO, SOD, CAT, and GST) and inflammation pathways [10]. Additionally, BCP promoted apoptosis in liver cancer cells via generating ROS, leading to mitochondrial membrane potential disruption and caspase-3 activation [24]. Moreover, the pro-apoptotic marker, Bax, was upregulated, and the anti-apoptotic markers were downregulated in dexamethasone-resistant human MM cell lines after being treated with BCP [49]. In parallel with that, BCP induced ROS generation at a 20 µM concentration in MG-63 cells, which was associated with mitochondrial apoptosis through increasing the levels of caspase-3 and Bax and the downregulation of Bcl-2 [42].

In line with that, BCP induced apoptosis through reducing HSP60, survivin, and other markers, along with increasing p21 expressions [6]. Apparently, BCP was able to suppress STAT-3, mTOR, and AKT protein expressions and thus inhibiting cytotoxicity in T24 and 5637 cells [8]. Moreover, BCPO was reported to be a safe anti-proliferative agent through induction of apoptosis PC-3 cells but with low toxicity towards normal cells [26]. Conclusively, besides involving in direct induction of apoptosis and enhancement of cell cycle arrest in cancer cells, Beta-Caryophyllene appears to synergistically enhance chemo-sensitization when co-supplemented with other drugs. However, interestingly, those BCP’s doses that can kill cancer cells can provide cryoprotection for normal cells.

## 5. Beta-Caryophyllene Reduces Angiogenesis and Cancer Cells’ Ability to Invade and Metastasize

After being transformed into malignant tumors, cancer cells ignore growth-inhibiting signals, resist apoptosis, and enhance angiogenesis to invade other tissues [50]. Angiogenesis is a critical process required for cancer progression to enable tumor growth and metastasis through the formation of new blood vessels. This occurs due to the less oxygen and nutrient contents at the tumor microenvironment, and thus, tumor cells may secrete the proangiogenic factor and the vascular endothelial growth factor (VEGF), the main driver of blood vessel growth. The latter can target the tyrosine kinase VEGF receptor 2 (VEGFR2) located at endothelial cells’ surfaces to promote angiogenesis [51,52]. Related to that, BCP has shown significant anti-angiogenic and then anti-metastasis properties through blocking migration of endothelial Cells, which is crucial for forming new blood vessels and suppressing endothelial cells from being organized into a network or capillary-like structures [6]. Additionally, BCP was found to inhibit the secretion of VEGF from human umbilical vein endothelial cells (HUVECs) leading to the reduction in both the cell migration and tube formation. However, as manifested using the rat aortic ring assay and chorioallantoic membrane assay, BCP blocked stages required new vessel formations such as vascular sprouting and vessel density [6]. Another attractive related issue is the antiangiogenic effect of BCP via CB2 receptor-driven VEGF inhibition and preventing the changes in the tumor microenvironment. That has been highlighted recently, where conditioned media (CM) from hypoxic lung cancer cells (A549 and H358), treated with BCP, showed inhibitory effects on VEGF secretion and HUVEC tube formation. The BCP inhibitory effects were CB2 receptor dependent [5]. That agrees with what has been reported earlier [53], where BCP-oxide was found to reduce STAT3 level in MM, prostate and breast cancer cell lines via upregulation of the tyrosine phosphatase, SHP-1, and by inhibiting the kinases, JAK1/2, and their downstream target, STAT3. Downregulation of STAT3 may inhibit the secretion of its downstream targets, including VEGF, leading to the suppression of angiogenesis [53]. BCP enhances potent inhibition of lymph node metastasis solid tumor growth of melanoma cells [54]. Similarly, BCP-oxide reduced tumor growth and inhibited metastasis through suppression of the signaling cascades of STAT3, PI3K/AKT/mTOR/S6K1 and ROS-mediated MAPKs activation. In the meantime, BCPO managed to block TNFα-NF-κB axis activation in a wide variety of cancer cell types to enhance apoptosis and suppresses invasion [47]. Another example reflecting the effective role of BCP in inhibiting cancer progression and metastasis is its effect on the regulators of the tumor microenvironment, the Matrix metalloproteinases (MMPs) that allow tumor cells to spread over the surrounding tissues in various malignancies but are blocked by MMPs inhibitors. In this regard, BCPO suppresses metastasis by prohibiting the secretion of MMP-2, p-p38 and p-ERK [15]. As seen from the findings above, Beta-Caryophyllene can efficiently inhibit angiogenesis and the cancer cells’ ability to invade and metastasize

## 6. Chemo-Sensitization Properties of Beta-Caryophyllene in Cancer Treatment

Chemotherapy is the main regimen used to treat several types of cancer. However, cancer cells may develop resistance to chemotherapy (chemoresistance) through mechanisms such as the efflux of drugs, mutation of drug targets, DNA-repair enhancement, or altered cell survival pathways. Therefore, it is important to apply strategies that can improve the cancer cells’ chemo-sensitization by using agents or modifying conditions leading to the enhancement of the efficacy of chemotherapeutic drugs. Out of these strategies, combination therapy can be applied using two or more drugs, where one agent can sensitize or enhance the activity of another by modulating one or more of the above mechanisms. In a study from 2007, BCP was suggested to promote drug accumulation by using different ways to potentiate the activity of anti-cancer drugs such as alpha-humulene or paclitaxel on human and breast cancer cell lines [45]. Ten years later, BCP and its oxides were hypothesized as possible potential chemo-sensitizing agents that can be used in combination with doxorubicin to modulate it in normal cells and to reduce the multidrug resistance phenomenon development in different drug-resistant cell lines [16]. A complex network mediated by BCP was proposed, in which STAT3 was suggested to act as an effector downstream enhancing doxorubicin-sensitivity of cholangiocarcinoma cells and increase the IC50 in nonmalignant cholangiocytes suggesting BCP as a dual-acting agent chemo-sensitization and chemoprevention [44]. On the other hand, combination therapy enabled BCP to promote apoptosis in A431 and HaCaT cells mediated by mitochondrial membrane potential loss, release of cytosolic cytochrome c, increase in Bax/Bcl-2 ratio, and enhance caspases and PARP cleavage [17].

## 7. Signaling Mechanisms Involved in BCP Enhancing Chemo-Sensitization Alone or in Combination in Cancer Therapy

**Figure 1:**
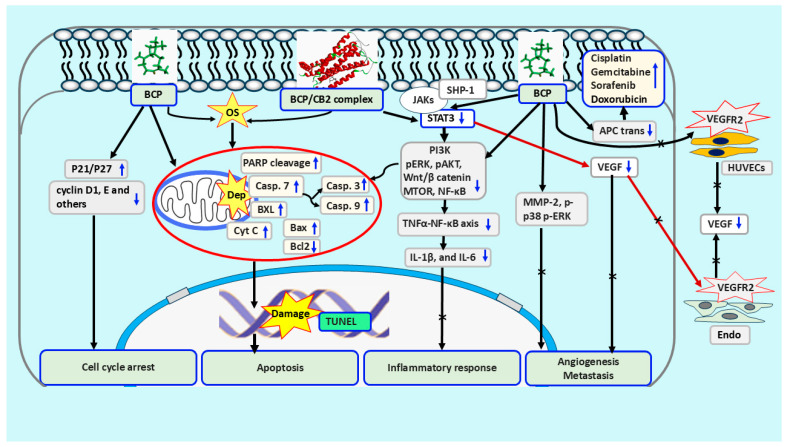
Signaling mechanisms involved in BCP-enhancing chemo-sensitization alone or in combination cancer therapy. ATP-binding cassette transporters (ABC trans), oxidative stress (OS), tyrosine kinase VEGF receptor 2 (VEGFR2), vascular endothelial growth factor (VEGF), endothelial cells (Endo), depolarization of mitochondrial membrane (Dep). (1) BCP induced cell cycle arrest through upregulating p21 and p27 and downregulating cyclin D1 and E and other kinases. (2) BCP induced apoptosis in cancer cells via direct dysregulation of the free radicals/antioxidants balance, and through increasing the levels of cleaved PARP, caspases and Bax, and the downregulation of Bcl-2, directly by interaction with CB2. (3) BCP can suppress STAT3 activation or inhibit its phosphorylation via SHP1 to reduce NF-κB and PI3K/AKT/mTOR and other cell cycle and apoptosis regulatory molecules, and, thus, proinflammatory cytokines, to inhibit inflammation and induce apoptosis in cancer cells; this can occur through interaction with CB2. (4) BCP blocks migration of endothelial cells by inhibiting the secretion of VEGF, and thus blocking the activity of the tyrosine kinase VEGFR2 located at endothelial cell surfaces (angiogenesis promoting receptors). (5) BCP can also prohibit the secretion of MMP-2, p-p38 and p-ERK besides inhibiting VEGF to efficiently prevent cancer cells from being able to invade and metastasize, that may occur through binding to CB2. (6) Through competitive binding to APC transporters, BCP reduces drug expulsion, increasing intracellular drug accumulation, and thus enhancing chemo-sensitization.

Based on the currently available data, when supplemented alone to cancer cell lines, BCP appears to use the same strategy used by the common chemotherapeutic drugs to disrupt DNA replication and DNA repair, dysregulate the free radical/antioxidant balance, induce cell cycle arrest and then apoptosis in these cells. Mechanistically, this occurs through modulation of signaling pathways required for cell survival and growth such as NF-κB and PI3K/AKT/mTOR, dysregulating the antioxidant enzymes (SOD, CAT and GPx) and ROS, as well as upregulating p21 and p27 and downregulating Wnt/β-catenin pathway and cyclin D1 and E, and other cyclin-dependent protein kinases (summarized in Figure 1). Beta-Caryophyllene was reported to inhibit the central to the regulators of inflammation, NF-κB and MAPK pathways, leading to a decrease in pro-inflammatory cytokine (TNF-α, IL-1β, and IL-6) production. Moreover, studies tried to understand the reasons for the synergistic role of BCP in combined therapy (listed in Table 3) by investigating the involved mechanisms and signaling molecules. A summarized overview of BCP’s downstream effects in the context of cancer, particularly in combination therapies, are discussed below (overviewed in Figure 1).

### 7.1. The Lipophilic Nature of BCP and Cancer Treatment

Beta-Caryophyllene is a highly lipophilic molecule which can interact with and integrate into lipid bilayers of cell membranes. In this regard, BCP was suggested to accumulate in the cancer cell membrane, altering the cells’ permeability, leading to the accumulation of anticancer drugs, and consequently strengthening the drugs’ activity. Thus, BCP can facilitate the passage of a drug such as paclitaxel through the cell membrane to potentiate the later anticancer activity [45]. In the meantime, the lipophilic nature of BCP and its ability to cross lipid bilayers are crucial for its interaction with cellular receptors, including CB2 receptors of cancer cells. As a result of binding to BCP, the CB2 receptor-mediated signaling pathways are triggered. In parallel with that, and probably because of its lipophilic nature, BCP was reported to interfere with ABC-mediated transport. In agreement with that, Di Sotto and colleagues [55] showed that, in spite of its potentially reactive chemical structure, BCPO is strongly absorbed through the membrane bilayer an thus interferes with the ABC transporters and reduces chemoresistance.

### 7.2. Selective Interaction of Beta-Caryophyllene with CB2 and Cancer Suppression

Besides its direct effect on cancer suppression, BCP can interact selectively with the body’s CB2 receptor activation by inhibiting the production of pro-inflammatory cytokines, helping to create an anti-tumor immune environment that suppresses pro-inflammatory cytokines (e.g., IL-6, TNF-α) and promotes anti-inflammatory mediators to inhibit tumor initiation and progression [28]. Related to that, in glioblastoma, BCP was reported to work as a tumor suppressor, regulating the CB2 receptor that activates PPARg and inhibits NF-κB and TNF-a, and subsequently reduces Jun N-Terminal Kinase (JNK) expression [19]. In the meantime, the BCP inhibitory effects on VEGF production, tube formation, and new blood vessels formation in animal models were CB2-receptor-dependent [5]. Additionally, marked cytotoxic effects of BCPO, in triple negative breast cancer MDA-MB-468 cells, were found to be mediated by CB2 receptor activation [56]. Additionally, it inhibited tumor growth by activating CB2 receptors and modulating inflammatory pathways [29].

### 7.3. Virtual Interaction of Beta-Caryophyllene with Cancer Involved Signaling Molecules

Molecular docking investigations have shown that BCP can interact with cyclin-dependent kinase CDK6, at four binding sites with a high (−7.8 kcal/mol) and stable energy [3]. Also, the direct interaction of BCP with P-gp was checked by molecular docking and dynamic simulation, where BCP interacted with active residues next to the ATP binding domain of P-gp. BCP showed a higher inhibition potential and stronger binding, as calculated by MM-PBSA [23]. This has been confirmed using in vitro models and described above. Moreover, BCP binds into the hydrophobic region of CB2 at the water-accessible cavity. The putative binding site of CB2 receptor ligands is located at sites crucial for the captor activity [56]. On the other hand, virtual interaction approach on BCPO indicated its binding affinity to anti-apoptotic proteins Bcl-2 and Mcl-1, which are often upregulated in various cancers [57]. However, further studies are required to understand the binding affinity of BCP on various signaling molecules involved in cancer progression.

### 7.4. STAT3 Works as a Downstram Effector of Beta-Caryophyllene Regulating a Complex Network in Cancer

Beta-Caryophyllene upstream effect on the JAK1/STAT3-signaling pathway was reported in several cancer studies [11,12,13,42,53,58]. For instance, BCP was found to induce apoptosis and initiate an inflammatory response via ROS and the JAK1/STAT3-signaling pathway in MG-63 cells treated with BCP [42]. This occurs due to activating signaling molecules that regulate inflammation, oxidative stress, and apoptosis, making BCP a promising therapeutic candidate in various pathological conditions, including cancer (Figure 1). Additionally, BCPO inhibited STAT3 activation in MM, prostate and breast cancer cell lines. The suppression was mediated via the suppression of the activity of the upstream kinases JAK1/2 and c-Src. Indeed, BCPO promoted the expression of the tyrosine phosphatase, SHP-1, which is associated with the reduction of the constitutive activation of STAT3. Interestingly, SHP-1 gene deletion (siRNA) inhibited the ability of BCPO to suppress STAT3 activation, resulting in the induction of apoptosis and abolition of the invasive potential of cancer cells [53]. In agreement with that, BCP was able to increase the cigarette smoke condensate-induced death in MDA-MB-468 cells, while it prohibited cell recovery, autophagy activation and cell migration through the deactivation of STAT3, representing a key mechanism of the chemoprevention by BCP [13]. The aggressive group of biliary tract tumors, CCAs, is characterized by multidrug resistance, late diagnosis, and poor outcomes. Trying to identify effective therapeutic protocol for CCA, the tumor antiproliferative activity was investigated using CCA cells and nonmalignant H69 cholangiocytes treated with doxorubicin combined with BCP, where BCP was able to synergize the cytotoxic effect of the low dose doxorubicin in CCA cells, a cytoprotective effects of BCP on the nonmalignant cells were observed. Interestingly, STAT3 was suggested to be an effective player in a complex network organized by BCP that enhanced doxorubicin-sensitivity of cholangiocarcinoma cells and decreased the toxicity in nonmalignant cholangiocytes, thus strengthening the idea of proposing BCP as a dual-acting, chemo-preventive and chemo-sensitizer, BCP as a dual-acting compound, a chemo-preventive and a chemosensitizer [44]. In this regard, research indicated that BCP can suppress STAT3 activation via inhibiting its phosphorylation at Tyr705, possibly through interference with upstream kinase JAK2, leading to downregulating STAT3 target genes such as Bcl-2 and cyclin D1 [58], Similarly, it was found that the STAT-3, mTOR, and AKT protein expressions were suppressed by BCP in T24 and 5637 bladder carcinoma cells leading to inhibition of cancer progression and migration [8]. Conclusively, STAT3 seems to be one of the major downstream effectors of BCP in regulating a complex network, including NF-κB and PI3K/AKT/mTOR and other cell cycle and apoptosis regulatory molecules in cancer.

### 7.5. BCP’s Effects on Transporters in Cancer in Relation to Its Synergetic and Chemosensitization Properties

As shown above, BCP can enhance chemosensitization in cancer by targeting ABC transporters, the key mediators of MDR. This may occur by direct inhibition of P-glycoprotein (P-gp/ABCB1) and multidrug resistance-associated protein 1 (MRP1/ABCC1), which are overexpressed in resistant tumors to efflux chemotherapeutics such as paclitaxel and doxorubicin. Through competitive binding to these transporters, BCP reduces drug expulsion, increasing intracellular drug accumulation and restoring apoptosis in resistant cancer cells [11,16,23,24]. For instance, in paclitaxel-resistant ovarian cancer models, BCP suppressed P-gp activity by 60–70%, significantly enhancing tumor sensitivity to treatment [23]. Additionally, β-caryophyllene oxide (CRYO) enhanced chemosensitization in hepatocellular carcinoma by inhibiting ABCB1, MRP1, and MRP2 [24]. At non-toxic concentrations, CRYO markedly reduced BCRP-induced resistance to known substrate drugs [25]. The combination of sorafenib and BCP synergized the anticancer drug, especially in pancreatic Bx-PC3 adenocarcinoma cells in a similar trend, but with a slightly lower efficacy, relative to standard ABC transporter inhibitors. The synergistic effect was associated with a modulation of MDR1 (or Pgp) and MRP transporters, both at the gene and protein levels; moreover, activation of the STAT3 cascade and cell migration appeared significantly affected, suggesting that the STAT3/ABC-transporter axis finely regulated efficacy and chemoresistance to sorafenib, thus appearing as a suitable target to overcome the drawbacks of sorafenib-based chemotherapy in hepato-biliary-pancreatic cancers [11].

## 8. Conclusions

Beta-Caryophyllene seems to be a promising potential drug in treating cancer when being supplemented in combination with drugs such as cisplatin, gemcitabine, and sorafenib. The modulation of these transporters by BCP may enhance the efficacy of chemotherapy by overcoming drug resistance mechanisms and improving drug bioavailability in cancer cells. Importantly, BCP’s selective antioxidant activity—activating Nrf2 in normal cells while increasing ROS in cancer cells—minimizes off-target toxicity, making it a promising adjuvant to overcome transporter-driven chemoresistance. BCP’s dual role in chemosensitization and toxicity mitigation, in addition to working as an angiogenesis inhibitor, positions it as a translational candidate for refractory cancers. Since BCP has not been yet clinically investigated, clinical trials are really needed to validate its potential clinical therapeutic effect.

## Figures and Tables

**Table 1:** Cancers potentially treatable with Beta-Caryophyllene.

Cancer Type	Mechanism of Action	Reference
Lung Cancer	Enhances cisplatin efficacy by promoting apoptosis and A549 cell cycle arrest	[3]
	Induces apoptosis and suppresses proliferative activity of lung cancer cells	[4]
	Induces G1 cell cycle arrest by dysregulating cyclins and other molecules	[2]
	Suppresses angiogenesis via modulating the secretome in hypoxic lung cancer cells	[5]
	Inhibits NSCLC growth to increase their apoptotic rate	[20]
Glioblastoma	Inhibits cell proliferation via direct targeting of CB2 receptors	[19]
	Works as a potential radiosensitizer for improving RT outcomes by inhibiting DNA repair, inducing apoptosis, and suppressing anti-apoptotic and survival pathways	[18]
Colorectal Cancer	Inhibits angiogenesis via suppressing the vascular endothelial growth factor (VEGF) signaling, reduces oxidative stress, and promotes apoptosis	[6]
	Activates initiator caspases via disruption of the mitochondrial membrane potential	[21]
	Inhibits the action of mono-ADP-ribosyltransferase 1 (ART1) by targeting nuclear factor-κB	[22]
Ovarian Cancer	Induces S-phase arrest in ovarian OAW 42 cells that display a high rate of apoptosis mediated by PARP cleavage and active caspase-3	[7]
Liver and Pancreatic Cancer	Enhances chemosensitization in liver, biliary, and pancreatic cancer cells treated with Sorafenib/doxorubicin using the STAT3/ABC transporter axis	[11,23,24]
	Enhances liver cancer cells apoptosis via ferritinophagy-mediated ferroptosis mechanisms	[9]
	Enhances sensitization of cholangiocarcinoma cells to chemotherapy via BCRP inhibition	[25]
	Suppresses tumor incidence by downregulating inflammation and inducing caspase-3-mediated apoptosis in a hepatocellular carcinoma	[10]
Prostate Cancer	Enhances mitochondrial membrane depolarization and thus activates caspase-7 dependent apoptosis	[26]
Breast Cancer	Enhances sensitization and promotes the cigarette smoke condensate (CSC)-induced apoptosis in MDA-MB-468 cells, mainly by triggering oxidative stress and inhibition of STAT3	[13]
	Enhances cholesterol biosynthesis transcriptional upregulation in breast cancer cells	[27,28]
	Combines therapy of DOX with BCPO synergistically and enhances the anti-proliferative effect on MDA-MB-231 breast cancer cells	[12]
	Inhibits tumor growth by activating CB2 receptors and modulating inflammatory pathways	[29,30]
Bladder Cancer	Inhibits bladder cancer cell proliferation and induces apoptosis via deregulation of STAT-3/mTOR/AKT signaling	[8]

**Table 2:** Summary of the IC50 values of Beta-Caryophyllene in normal and cancer cell lines.

Cell Type	IC50 (μM)	Reference	Notes
**Normal Cells (Control)**			
Skin Fibroblasts	>100	[33]	Low cytotoxicity in normal cells
Peripheral Blood Mononuclear Cells (PBMCs)	>100	[40]	Minimal toxicity in immune cells
Normal Human Fibroblasts	>200	[7]	Highest IC50 in fibroblasts
Normal Human Lymphocytes	<100	[41]	Safe at low concentrations
Normal Fibroblasts (comparison)	195 μg/mL	[42]	Paired with hepatoma cells (see below)
3T3-L1 (Mouse Embryonic Fibroblasts)	>100	[43]	Requires ≥ 2× higher dose vs. cancer cells
RGC-5 (Retinal Ganglion Cells)	>100	[43]	Requires ≥ 2× higher dose vs. cancer cells
**Cancer Cells**			
Bladder Cancer (T24 & 5637 cells)	40 µg/mL (~64 μM)	[8]	Dose- and time-dependent cytotoxicity
Colon Cancer (HCT-116)	19	[6,42]	Most sensitive cancer line
Bone Cancer (MG-63)	20	[6,42]	IC50 within typical range (19–64 μM)
Pancreatic Cancer (PANC-1)	27	[6,42]	IC50 within typical range (19–64 μM)
Hepatocellular Carcinoma (HepG2)	44.7 µg/mL (~72.8 μM)	[23]	Pure compound administration
Breast Cancer (MDA-MB-468)	19.2 µg/mL (~31.5 μM)	[23]	Pure compound administration
Cervical Cancer (ME-180)	Requires higher doses for toxicity	[44]	≥2× lower sensitivity vs. normal cells
Triple Negative Breast Cancer (MCF-7)	Requires higher doses for toxicity	[44]	≥2× lower sensitivity vs. normal cells
Hepatoma Cells	63.7 μg/mL	[43]	One-third of the value required to induce toxicity in fibroblasts, under same conditions.

**Table 3:** Therapeutic drugs used in combination treatment of cancer with Beta-Caryophyllene.

Chemotherapeutic Agent	Mechanism of Action	References
Paclitaxel	Reduces tumor growth by increasing the accumulation of Paclitaxel and facilitating its passage through the membrane to potentiate its anticancer activity. The paclitaxel anticancer activity, in colorectal adenocarcinoma cell line, increased to about 10-fold when combined with BCP.	[45]
Doxorubicin	Enhances tumor suppression by increasing the drug accumulation; inhibiting the efflux pumps; reducing P-gp; facilitating cycle arrest; and inducing STAT3 activation.	[12,16,21,23,46]
Cisplatin	Regulates cell cycle and apoptosis signaling molecules, and targets the placental growth factor gene and MAPK signaling pathways, and inhibits ABC proteins (such as MRP1, MRP2, and BCRP) that are very relevant in chemoresistance	[3,25,43]
5-Fluorouracil (5-FU)	Enhances apoptosis and reduces tumor proliferation by disrupting the mitochondrial membrane potential and activating initiator caspases	[21]
Alpha-Humulene	Reduces tumor growth and enhances BCP’s therapeutic effects.	[47]
Isocaryophyllene	Potentiates the anticancer activity by facilitating drug accumulation within the cells.	[47]
Gemcitabine	Improves tumor suppression, reduces drug resistance, and inhibits the ABC proteins	[21]
Sorafenib	Enhances tumor suppression and reduces chemoresistance to sorafenib through the modulation of AMRP and MDR1 (or Pgp) transporters and regulation of STAT3/ABC-transporters axis.	[11,16,25]

## Data Availability

No new data were created or analyzed in this study. Data sharing is not applicable to this article.
